# Long−Term Follow−Up of a Pseudohypoparathyroidism Type 1A Patient with Missense Mutation (Pro115Ser) in Exon 5

**DOI:** 10.4274/jcrpe.v2i2.85

**Published:** 2010-05-07

**Authors:** Şenay Savaş Erdeve, Merih Berberoğlu, Zeynep Şıklar, Olcay Evliyaoğlu, Olaf Hiort, Gönül Öcal

**Affiliations:** 1 Ankara University School of Medicine, Department of Pediatrics, Division of Pediatric Endocrinology, Ankara, Turkey; 2 Kırıkkale University School of Medicine, Department of Pediatrics, Division of Pediatric Endocrinology, Ankara, Turkey; 3 University of Lubeck, Department of Pediatrics, Division of Pediatric Endocrinology and Diabetes, Lubeck, Germany; +90 312 595 67 91senaysavas@yahoo.comAnkara University School of Medicine, Department of Pediatrics, Division of Pediatric Endocrinology, Ankara, Turkey

**Keywords:** Pseudohypoparathyroidism type Ia, Albright’s hereditary osteodystrophy, mutation

## Abstract

Pseudohypoparathyroidism (PHP) refers to end−organ resistance that primarily impairs the renal actions of parathyroid hormone (PTH). The patients with PHP type Ia (PHP−Ia), one of the 4 types of PHP, show resistance to other peptide hormones as well as clinical features of Albright hereditary osteodystrophy (AHO), a constellation of short stature, obesity, brachydactyly, ectopic ossifications, and/or mental retardation. Here we report clinical follow−up for a long−term period in a PHP−Ia case who had a missense mutation leading to the substitution of proline by serine (Prol115Ser) in exon 5 which has been reported previously in only two patients. An 11−year−old boy applied for hand spasm to our hospital. On physical examination, he had short stature, round−shaped face and brachydactly. Laboratory evaluation revealed PTH and TSH resistance. Molecular genetic analysis of the GNAS gene revealed a P115S substitution. The patient was followed up for 13 years. Normocalcaemia was achieved with reduced doses of calcitriol (0.25 μg/day) and calcium supplements (40 mg/kg/day). Daily requirement for levothyroxine supplementation was still high (2.3 μg/kg) to achieve euthyroidism. His pubertal development was Tanner stage V and he has no gonadotropin resistance. To our knowledge, this is the first report concerning long−term follow−up of this rare mutation. We believe that despite the genetic heterogeneity of AHO, phenotype/genotype correlations of this kind of rare mutations may help to understand progress of the disease.

**Conflict of interest:**None declared.

## INTRODUCTION

Pseudohypoparathyroidism (PHP), also known as Albright’s hereditary osteodystrophy (AHO), refers to a heterogeneous group of rare metabolic disorders characterized by hypocalcemia and hyperphosphatemia due to parathyroid hormone (PTH) resistance. PHP is associated with a constellation of physical features which include short stature, obesity, round face, brachydactyly, and subcutaneous ossifications. PHP type−I (PHP−I), a term used to describe a condition in patients who show a blunted nephrogenous cAMP response to exogenously administered bioactive PTH, can be further classified into three different subtypes based on the presence of additional endocrine abnormalities, deficiency of the α−subunit of the stimulatory G protein (Gsα), and dysmorphic features of AHO (1). In addition to resistance to PTH, patients with PHP−Ia may have resistance to other hormones, such as thyrotropin (TSH) and gonadotropins that stimulate cAMP formation in the target cells by interacting with receptors coupled to Gs, the stimulatory protein of adenylate cyclase. These patients typically carry heterozygous inactivating mutations in one of the 13 GNAS exons encoding Gsα (2).

Although several studies investigated the phenotypic heterogeneity of PHP, minimal information exists on the temporal progression of the hormone resistance (1, 3, 4). Here we report long−term clinical follow−up findings in a PHP−Ia patient, who had a missense mutation leading to the substitution of proline by serine (Prol115Ser) in exon 5, a mutation which has been reported in only two patients since 1998 (5, 6).

## CASE REPORT

**Figure 1:**
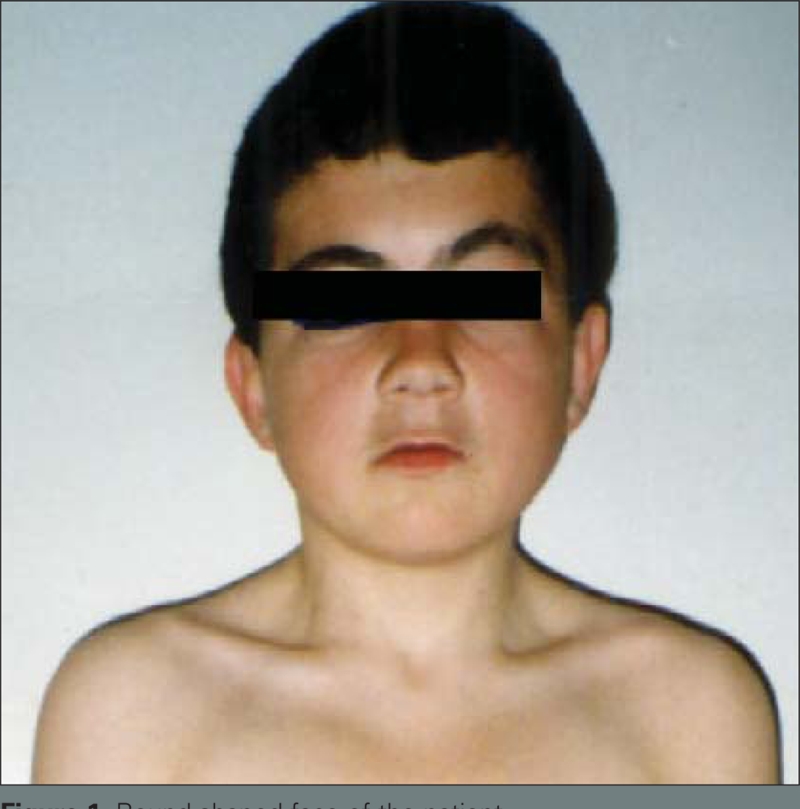
Round−shaped face of the patient

**Figure 2:**
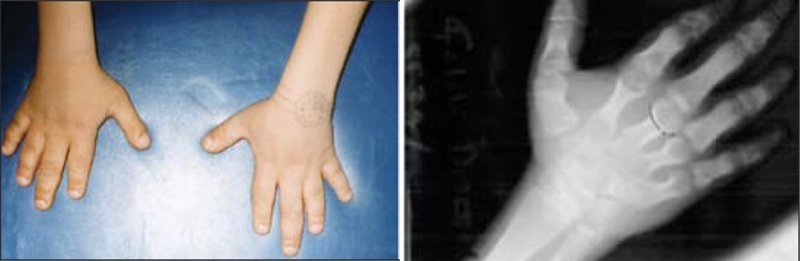
Brachydactyly and metacarpal shortening in the patient

An 11−year−old boy was admitted to our department because of hand spasm. He was the first son of first−degree consanguineous parents and was delivered after an uncomplicated pregnancy. The family did not report any sign of disease during infancy. On physical examination, the patient weighed 29.5 kg (3−10^th^ percentile) and his body mass index was 18.7 (25−50^th^ percentile). Short stature (height 125.5 cm, <3^rd^ percentile), round−shaped face (Figure 1) and brachydactyly were recorded as pathological findings. His total hand length was 12.7 cm (3_rd_ percentile) and his palm length was 8 cm (<3^rd^ percentile) (7) (Figure 2). There was no family history of hypocalcemia or brachydactyly. The patient showed a pubertal development consistent with Tanner stage II. Radiological assessment showed a normal bone age and brachydactyly (short metacarpals, especially in the 4_th_ and 5_th_ fingers and short proximal phalanges, especially of 2_nd_, 3_rd_ and 5_th_ fingers) (Figure 2). Based on these clinical findings, a tentative diagnosis of AHO was considered.

**Figure 3:**
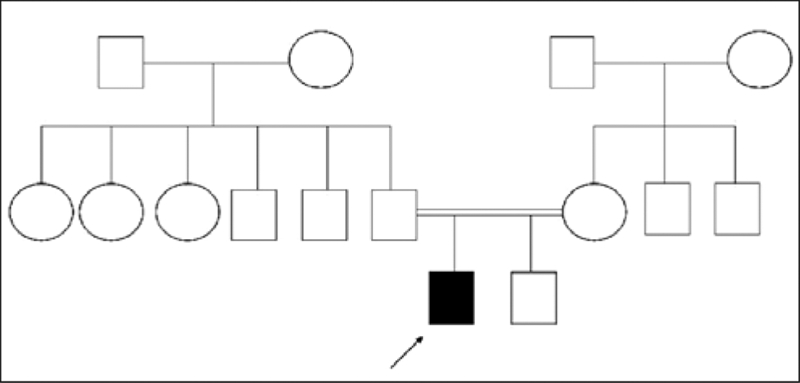
Pedigree of the family with PHP−Ia patient

Laboratory tests revealed reduced serum calcium (7.6 mg/dL, N: 8−10.2 mg/dL), elevated serum phosphorus (7 mg/dL, N: 2.7−4.5 mg/dL) and markedly elevated serum parathormone (6.8 ng/dL, N: 0.01−2.7 ng/dL) levels. Serum TSH level was high (16.2 mIU/ml, N: 0.5−5 mIU/ml). Serum free thyroxine (fT4) and free triiodothyronine (fT3) levels were 12 pmol/l (N; 10−22 pmol/L) and 4.7 pmol/l (N; 3.5−5.5 pmol/L), respectively, indicating subclinical hypothyroidism. Anti−thyroid antibodies were absent and thyroid ultrasound findings were normal. Growth hormone (GH) response to insulin tolerance test was normal (14.5 ng/mL). Renal ultrasound findings revealed no nephrocalcinosis. Cranial computerized tomography (CT) scan demonstrated no basal ganglion calcification. These results, including resistance to TSH and PTH, were consistent with a diagnosis of AHO (PHP type−Ia). Genomic DNA was extracted with phenol−chloroform method from peripheral blood leukocytes. Sequence analysis of the GNAS gene identified a missense mutation leading to the substitution of proline by serine (Pro115Ser) in exon 5. The family pedigree is shown on Figure 3.

For treatment, vitamin D (1,25−(OH)_2_D_3_) in a dose of 1 μg/day and 40 mg/kg/day elementary calcium together with a low−phosphorus diet were started. This treatment regimen resulted in normalization of calcaemia and phosphataemia. The patient also received L−T4 treatment in a dose of 1.6 μg/kg/day.

**Figure 4:**
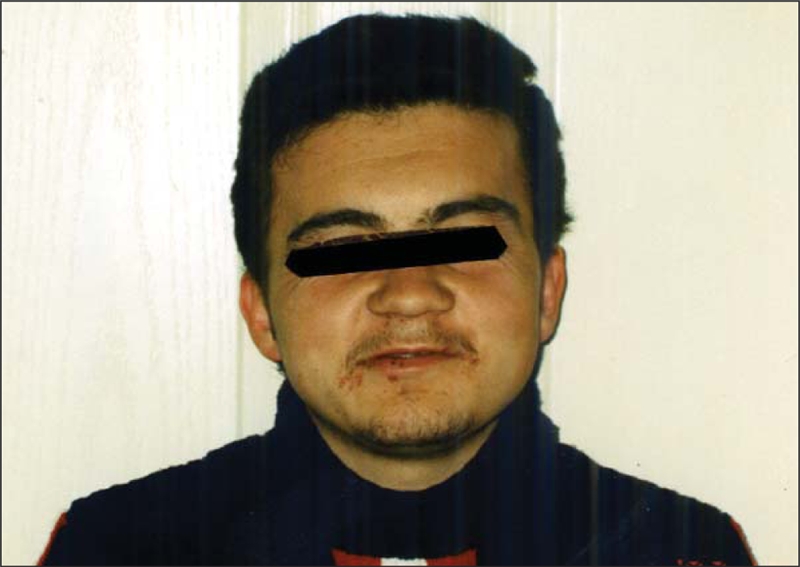
Facial appearance of the patient at 24 years of age

During the 13−years of follow−up, the patient showed no obesity or cutaneous calcifications. At the age of 24, his height was 146 cm (−4.37 SD), and his weight was 52.4 kg. Body mass index was 24.6. His daily requirement for L−T4 supplementation was still high (2.3 μg/kg). Normal calcaemia was achieved with reduced doses of calcitriol (0.25 μg/day) and calcium supplements (40 mg/kg/day). His pubertal development was at Tanner stage V and he had no gonadotropin resistance (Figure 4).

## DISCUSSION

The human Gsα gene (GNAS1) contains 13 exons encoding the Gsα and is located at 20q13.11. Heterozygous loss of function mutations in GNAS1 have been identified in the majority patients with PHP−Ia (2). The missense mutation in exon 5 resulting in a proline to leucine change in codon 115 has been described before by Ahrens et al (8) and de Sanctis et al (9). Sequence analysis of our patient identified a missense mutation leading to the substitution of proline by serine (Pro115Ser) in exon 5, a finding which was previously reported in only two cases (5, 6). The Pro115Ser mutation resulted from a C to T transition (CCC⇒TCC). The Pro115Ser mutation is predicted to disrupt the highly conserved domain of Gsα that interacts with adenylate cyclase (10).

Linglart et al (11) investigated the role of the different mutations of GNAS in various clinical phenotypes observed (genotype−phenotype correlation). They could not find clinical or biological heterogeneity between the PHP−Ia patients with and without GNAS gene mutation. However, they demonstrated that the proband with a mutation resulting in a premature stop codon and truncated protein had a severe multihormonal resistance phenotype. The phenotype appeared to be milder in two cases and severe in one with substitutive mutations. A 41−year−old patient with R280K mutation, who had been followed for 29 years was shown to have isolated PTH resistance without TSH resistance, whereas the second mild case had AHO and TSH resistance but compensated PTH resistance. The patient who showed severe clinical symptoms was diagnosed in his first year of life, had TSH resistance and developed PTH resistance similar to our patient (11). Ahmed et al (5) reported PTH and TSH resistance in a 6−year−old girl who manifested with brachydactyly and subcutaneous calcification and had Pro115Ser mutation. De Sanctis et al (9) reported the same resistance in a 12−year−old girl, who had brachydactyly, obesity, round−shaped face and short metacarpals but showed Pro115Leu mutation. The hormonal resistance primarily affects PTH. However, in PHP−Ia patients, due to the ubiquitous distribution of G proteins, multihormonal resistance occurs, impairing responses to TSH, and also pituitary and hypothalamic hormones (12). These patients have elevated TSH levels, indicating TSH resistance at the receptor−complex level. In the first years of life, hypothyroidism is the most common presenting symptom (3). Recent evidence indicates that the GNAS gene is incompletely imprinted in the thyroid, leading to variable presentation of the disease. PTH resistance shows a progressive trend and frequently is expressed later in childhood (13). In our patient, PTH resistance was associated with TSH resistance. On the other hand, the patient had spontaneous pubertal development and had no gonadotropin resistance. Gelfand et al (3) identified trends in presentation and clinical disease progression in 12 patients with PHP/AHO/multi−hormone resistance. Of the five patients evaluated for hypogonadism, one had gonadotropin resistance together with PTH and TSH resistance. This patient was a 17−year−old female and was followed for 11 years. However, genotype−phenotype correlation was not investigated (3).

The prevalence of short adult height in PHP−Ia is reported to be as high as 80% (14). Some of PHP children have GH deficiency secondary to absent response of GH to GHRH (15). Although our patient had short stature, GH deficiency was not detected in the evaluation. The final height SDS in our patient was calculated as −4.37 SDS.

To our knowledge, this is the first report of a long−term follow−up in a patient with this rare mutation. We believe that despite the genetic heterogeneity of AHO, investigation of phenotype/genotype correlations in this kind of rare mutations may help to understand the progress of the disease.
